# Remifentanil added to sufentanil-sevoflurane anesthesia suppresses hemodynamic and metabolic stress responses to intense surgical stimuli more effectively than high-dose sufentanil-sevoflurane alone

**DOI:** 10.1186/1471-2253-15-3

**Published:** 2015-01-18

**Authors:** Ingo Bergmann, Torsten Szabanowski, Anselm Bräuer, Thomas A Crozier, Martin Bauer, José Maria Hinz

**Affiliations:** Department of Anaesthesiology, Emergency and Intensive Care Medicine, University of Göttingen Medical School, Robert-Koch Str. 40, 37075 Göttingen, Germany

**Keywords:** Sternotomy, Stress response, Remifentanil, Intraoperative hypertension, Oxygen uptake

## Abstract

**Background:**

Even extremely high-doses of the potent opioid, sufentanil, cannot reliably suppress stress responses to intense surgical stimuli such as sternotomy. The chemically related opioid remifentanil with its different pharmacokinetics and binding affinities for delta- and kappa-opioid receptors might be more effective in attenuating these responses.

**Methods:**

ASA I-III patients scheduled for a surgical procedure with sternotomy under balanced anesthesia (sevoflurane and sufentanil 3 μg.kg^-1^ bolus, 0.017 μg.kg^-1^.min^-1^ infusion) were randomized into two groups. Patients in the study group were supplemented with remifentanil (2 μg.kg^-1^ bolus, 2–7 μg.kg^-1^.min^-1^ infusion) starting ten minutes before sternotomy. Heart rate, arterial blood pressures, cardiac index, ejection fraction, systemic vascular resistance index (SVRI), total body oxygen uptake (VO_2_) and electric dermal response were measured and compared between the groups.

**Results:**

62 patients were studied (study group 32, control group 30). Systolic and mean arterial blood pressures, SVRI, VO_2_ and skin conductance increased during sternotomy and sternal spread in the control group but not in the study group. Systolic blood pressure increase: 7.5 ± 19 mmHg vs. -3.4 ± 8.9 (p = 0.005); VO_2_ increase: 31 ± 46% vs. -0.4 ± 32%; incidence of systolic blood pressure increase greater than 15 percent: 20% vs. 3% (p = 0.035) (control vs. study group).

**Conclusion:**

High-dose remifentanil added to sevoflurane-sufentanil anesthesia suppresses the sympathoadrenergic response to sternotomy and sternal spread better than high-dose sufentanil alone.

**Trial registration:**

Clinical Trial number: DRKS00004327, August 31, 2012

**Electronic supplementary material:**

The online version of this article (doi:10.1186/1471-2253-15-3) contains supplementary material, which is available to authorized users.

## Background

Sympathoadrenergic responses to surgical trauma affect perioperative morbidity and mortality and are particularly undesirable in patients with ischemic or congestive heart disease. Increased blood pressure and oxygen consumption can cause myocardial ischemia and ultimately worsen the patients' perioperative prognosis [1]. High doses of opioid narcotics were shown to provide hemodynamic stability and suppress most reactions to surgical trauma [2–5]. But even sufentanil, administered at up to one hundred-fold the normal anesthetic dose, cannot reliably prevent the responses to sternotomy and sternal spread [6], a stimulus that is known to cause myocardial ischemia [7]. The reason for this is that the maximal effect attainable with sufentanil is insufficient. This effect cannot be increased by administering a even larger dose (ceiling-effect) as shown by the sigmoid plasma concentration-response curve [8, 9].

Remifentanil is chemically related to sufentanil [10], but differs from it in several aspects. Remifentanil has an extremely short context-sensitive half time [11] which allows it to be administered in very high doses during particularly stressful intraoperative phases without prolonging recovery time [10, 12]. Remifentanil also has different affinities for cardiac kappa and delta opioid receptors [13]. These receptors are thought to mediate the cardioprotective effect reported for remifentanil [13] and could also possibly be exploited to attenuate the cardiovascular responses that do not respond to the predominantly μ-opioid receptor activation of sufentanil. One possible disadvantage of remifentanil might be its reported link to opioid-induced hyperalgesia [14, 15].

We hypothesized that high-dose remifentanil given before and during sternotomy in addition to a balanced high-dose sufentanil-sevoflurane anesthesia could attenuate the cardiovascular and metabolic stress responses.

## Methods

### General

This prospective, randomized double-blinded study was conducted with the approval of our clinical research ethics committee (Universitätsmedizin Göttingen, Ethikkommission 14/2/11, on 29 March 2012). It was registered on August 31, 2012 with the German registry of clinical trials (Deutsches Register für klinische Studien (http://drks-neu.uniklinik-freiburg.de/drks_web/navigate.do?navigationId=trial.HTML&TRIAL_ID=DRKS00004327) under the trial number DRKS 00004327. The participating patients gave their written consent after having been informed by the principal investigator, Dr. Bergmann, both orally and with written information material.

### Patients

ASA I - III patients scheduled for surgery requiring sternotomy for cardiac surgery were eligible for inclusion. Exclusion criteria were emergency surgery or a preoperative hemoglobin concentration under 10 g dL^-1^. After having given written informed consent the patients were randomized to the control or the study group using a computer-generated list (http://www.randomizer.org). All patients had a balanced anesthesia with high-dose sufentanil and sevoflurane. In addition to these drugs, the patients in the study group were given remifentanil as a continuous infusion in the period before and during sternotomy (see below). Two investigators (IB and TS) performed all anesthetics.

### Patient preparations

Premedication consisted of oral midazolam 7.5 mg given on the ward 20 to 30 minutes before induction of anesthesia. In the operating room an infusion was established via a peripheral vein and the radial artery was cannulated. We attached forehead electrodes for measuring state entropy (S/5 monitor, GE Healthcare, Helsinki, Finland) and palmar Ag/AgCl_2_ electrodes for measuring skin conductance (Elektrosympathograph 1001, Ingenieurbüro Dr. Janitzki, Altenbeken, Germany [16]). After induction, a four-lumen central venous catheter and a pulmonary artery catheter were inserted; the latter was positioned with the inflated balloon in wedge position. The transesophageal echocardiography (TEE) probe was introduced and used to assess cardiac filling volumes and measure ejection fractions (fractional area change, FAC, in the transgastric mid short-axis view).

### Monitoring

Heart rate, systolic and mean arterial pressure (SAP, MAP), pulmonary artery pressure (PAP), central venous pressure (CVP), oxygen consumption (VO_2_), Surgical Pleth Index (SPI, see below), entropy (SE and RE) and skin conductance as an indicator of sympathetic nervous system activity were monitored continuously and the data stored for offline analysis by a researcher not involved in data acquisition and blinded to the patient's group. VO_2_ was measured by indirect calorimetry with the integrated metabolic monitor module of the respirator (Datex Ohmeda M-COVX). Plasma catecholamine concentrations were not determined because they are not reliable indicators of endogenous secretion in this setting, since many patients are routinely given catecholamine infusions during the pre-sternotomy phase, as was the case in the present study

Cardiac output (CO) was measured by thermodilution after intubation, at skin incision for vein harvesting, at sternotomy and ten minutes after sternum spreading. Ice-cold 0.9% NaCl was used as injectate. Pulmonary artery wedge pressure (PAWP) and mixed-venous oxygen saturation (SvO_2_) were also measured at these times. Cardiac (CI) and systemic vascular resistance indexes (SVRI) were calculated from the data. Ejection fractions were determined before and after sternotomy. At these time points sufficient crystalloid fluids had been infused to correct any pre-existing hypovolemia and normalize cardiac filling volumes in all patients.

The Surgical Pleth Index (SPI) is an indicator of the body's response to noxious stimuli and thus indirectly of the intensity of analgesia and was used to guide the administration of the remifentanil and sufentanil [12, 17, 18]. The SPI is a dimensionless number between 0 (no response) and 100 (strong response) that is calculated from the heart rate and the pulse-induced volume changes of the finger (photoplethysmography), which are registered by a fingertip sensor. Entropy correlates with the depth of hypnosis [19–21] and was used to adjust the end-tidal sevoflurane concentration. The electrodermal response (EDR) is the change in skin conductance in response to a stimulus and is correlated with the activity of the sympathetic nervous system [22]. A change in skin conductance occurring after the stimulus was recorded as a sympathetic response.

The study period ended and acquisition of hemodynamic and metabolic data was stopped after the last measurement following sternal spread.

Pain intensity was assessed with a numeric rating scale (NRS) from 1 to 10 during the first twenty-four hours in the intensive care unit by nursing staff blinded to the patient's group allocation. Opioid analgesic consumption data were extracted from the patients' charts.

### Anesthesia

Anesthesia was induced with sufentanil (150 μg bolus for patients under 80 kg; 200 μg for patients over 80 kg) and midazolam (3–6 mg titrated to hypnotic effect). Rocuronium (1 mg kg^-1^) was given to facilitate intubation of the trachea. The patients' lungs were ventilated with 60% oxygen in air in volume-controlled mode with a tidal volume of 6 ml kg^-1^ and PEEP 7 cmH_2_O. The respiratory rate was adjusted to keep end-tidal CO_2_ between 35 and 40 mmHg and sevoflurane was added in an initial age-adapted concentration of 0.5 MAC. The chosen sevoflurane concentrations were kept constant by the end-tidal control (ETC.) of the anesthesia machine (Aisys®, GE Healthcare).

Anesthesia was maintained in both groups with sevoflurane and a continuous infusion of sufentanil (0.017 μg kg^-1^ min^-1^). The end-tidal sevoflurane concentration was adjusted to keep SE below 60 and the difference between RE and SE below 10.

A second bolus injection of 50 μg sufentanil was given ten minutes before sternotomy. Immediately following the injection the remifentanil infusion was started in the study group with a 2 μg kg^-1^ loading dose and an initial infusion rate of 2 μg kg^-1^ min^-1^. The infusion rate was adjusted in steps of 0.1 μg kg^-1^ min^-1^ to keep the SPI value between 20 and 50. The infusion rate was also increased by 0.1 μg kg^-1^ min^-1^ if the SPI value increased suddenly by more than 10, even if the SPI was still within the target range. The remifentanil infusion was continued until the last measurement after sternotomy. Additional sufentanil was not administered in the control group because the plasma sufentanil concentrations were on the upper plateau of the concentration-effect curve and a further reduction of SPI could not be achieved with sufentanil.

Mean arterial pressure was kept between 60 and 80 mmHg. We increased the inspiratory sevoflurane concentration if MAP persisted in any patient at over 80 mmHg. The treatment MAP below 60 mmHg was based on cardiac index, SVRI and cardiac filling volumes and consisted of either the administration of dobutamine or norepinephrine or the infusion of additional fluids. Bradycardia was defined as a heart rate under 45 beats per minute and was treated with an infusion of dobutamine if associated with hypotension.

### Statistical analysis

The data were analyzed with the statistics program Statistica® (StatSoft Europe). Normal distribution was tested with the Kolmogorov-Smirnov test. Normally distributed data were described by mean and standard deviation, non-parametric data by median and range. Categorical data were given as percentages. ANOVA was used to test for changes over time between the groups. Student's *t*-test for paired samples was used to pairs of sampling points in individual groups. Mann–Whitney *U*-test was used for non-parametric data and Fisher's exact test for categorical data. A p-value equal to or less than 0.05 was considered statistically significant.

The primary end-point of the study was a hemodynamic response to sternotomy and sternal spread with the response defined, as in most similar studies, as a greater than 15% increase in systolic blood pressure. Secondary end-points were other indicators of increased sympathetic nervous system activity, i.e. change in skin conductance, increased cardiac output, increased systemic vascular resistance and increased oxygen consumption.

For calculating the group size required to give an adequately small Type II error (i.e. adequate statistical power) we referred to the average published response rate to sternotomy and sternal spread under high-dose sufentanil anesthesia in studies that used the same definitions as employed in the present study; this is approximately 35%. Two groups with 26 patients each would be required to detect a reduction of the response rate to 10% with a power of 75% and a statistical significance level of 5%. We chose to use two groups of 33 patients each to allow for possible dropouts.

## Results

**Figure 1:**
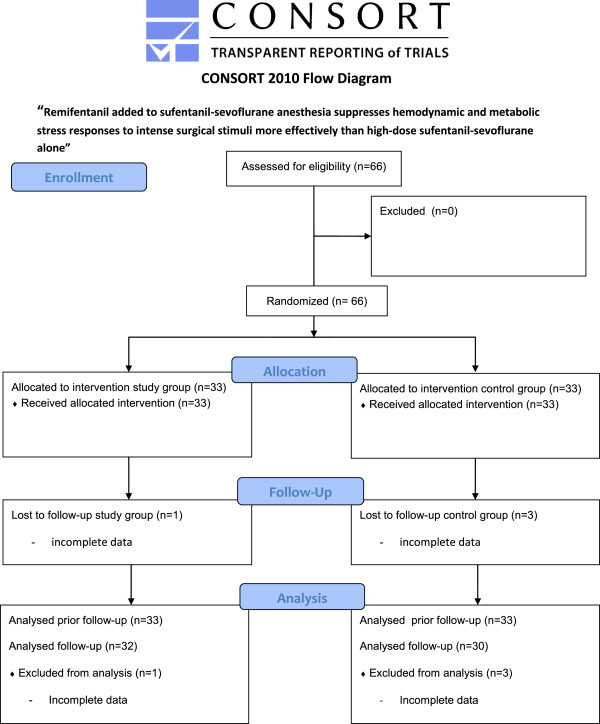
**CONSORT 2010 flow diagram.**

Sixty-six patients were recruited for the study. Four were excluded from the final analysis because of incomplete data sets or withdrawn consent (one in study group, three in control group) (Figure 1: CONSORT flow diagram, Additional file 1). The groups (study group n = 32; control group n = 30) did not differ with regard to age, height, body mass index, sex, or ASA classification. All patients were on chronic medication with beta-blockers (Table 1).

**Table 1:** **Demographic patient data and dosage and consumption of anesthetic drugs**

	Study group n = 32	Control group n = 30	p
BMI (kg m^-2^)	27.3 ± 4.8	28.9 ± 5.0	0.18
Male/Female (n)	19/13	24/6	0.18
ASA I/II/III (n)	0/0/32	0/0/30	1.0
Chronic beta-blocker therapy (n)	32/32	30/30	
**Remifentanil** (maximum infusion rate in μg kg^-1^ min^-1^)			
before sternotomy	2.0 [0.0 - 6.0]	-	n.a.
during sternotomy	3.0 [2.0 - 7.0]	-	n.a.
after sternotomy	2.5 [0.3 - 7.0]	-	n.a.
**Cumulative opioid doses before sternotomy**			
Sufentanil (μg)	345 ± 68	355 ± 45	0.5
Remifentanil (mg)	6.2 [1.1 - 22.1]	-	n.a.

(means ± standard deviation, median [range] or count).

n.a. not applicable.

The infusion rates and doses of anesthetic drugs are shown in Table 1. The total sufentanil dose administered from induction to sternotomy was the same in the two groups.

The SPI increased significantly in both groups during intubation. The patients in the study group were not receiving remifentanil at this time. During sternotomy and sternal spread, SPI increased significantly from 29 to 58 in the control group but did not change in the patients of the study group, to whom remifentanil was now being administered. The groups differed significantly at this time (see Table 2). State entropy values were within the target range in both groups, but were significantly lower in the study group after starting the remifentanil infusions. The groups did not differ with regard to the number of patients who showed a sympathetic response to intubation (evidenced by the electrodermal response and SPI), which occurred before the remifentanil infusion was started. But the number of patients with increased sympathetic nervous system activity (evidenced by the electrodermal response and SPI) in response to sternotomy was significantly lower in the study group (Table 2).

**Table 2:** **Values of surgical Pleth index and electrodermal response**

	Study group	Control group	p*
	n = 32	n = 30	
**SPI**			
Before surgery before Intubation	42 ± 14	42 ± 17	0.99
after Intubation	53 ± 14^†^	58 ± 17^†^	0.23
During surgery			
before sternotomy	35 ± 12	29 ± 18	0.13
during sternotomy	40 ± 15	58 ± 14^†^	<0.001
**EDR (n (%))**			
responders to intubation	12 (36%)	12 (39%)	0.86
responders to sternotomy	12 (37%)	24 (81%)	<0.001

(SPI = Surgical Pleth Index; EDR = electrodermal response, means ± standard deviation).

*Comparison of study vs. control group.

^†^Comparison before intervention vs. after intervention.

The courses of blood pressures and heart rate are shown in Table 3. Systolic and mean arterial blood pressure increased significantly less in response to sternotomy in the remifentanil study group. Sternotomy caused a greater than 15% increase in systolic blood pressure in six patients in the control group (20%) and only one (3%) in the remifentanil group (p = 0.035). Mean arterial pressure reacted similarly with a 63% response rate in the control group compared to 38% in the remifentanil group (p = 0.42).

**Table 3:** **Intraoperative course of heart rate and blood pressure**

	Study group	Control group	p
	n = 32	n = 30	
**Systolic arterial pressure** (mmHg)			
before intubation	108 ± 26	114 ± 19	0.17
after intubation	108 ± 27	115 ± 22	0.25
During surgery before sternotomy	100 ± 17	100 ± 14	0.99
during sternotomy	102 ± 13	115 ± 25 †	0.01
**Mean arterial pressure** (mmHg)			
before intubation	72 ± 15	78 ± 14	0.15
after intubation	74 ± 16	77 ± 14	0.35
During surgery before sternotomy	72 ± 9	76 ± 14	0.2
during sternotomy	70 ± 8	79 ± 13	0.001
**Heart rate** (min^-1^)			
Before Intubation	61 ± 15	60 ± 14	0.6
After Intubation	61 ± 12	61 ± 13	0.9
During surgery before sternotomy	54 ± 10	53 ± 8	0.9
during sternotomy	54 ± 10	54 ± 10	0.8
**Hemodynamic response to sternotomy and sternal spread**			
Patients with >15% increase in systolic pressure (n [%])	1 [3%]	6 [20%]	0.035
Average systolic blood pressure change	-3.4 ± 8.9	7.5 ± 19	0.005
Average mean arterial pressure change	-2.8 ± 8.2	5.9 ± 18	0.02

(means and standard deviation unless otherwise indicated).

Whole body oxygen consumption and systemic vascular resistance index increased significantly in the control group. The ejection fraction (fractional area changes) decreased significantly in the control group (Table 4).

**Table 4:** **Extended hemodynamic monitoring data**

	Measuring time points					
	Before sternotomy		During sternotomy		After sternotomy	
Parameter	Study	Control	Study	Control	Study	Control
PAPsyst (mmHg)	29 ± 9	29 ± 7	31 ± 8	33 ± 8	31 ± 6	31 ± 8
PCWP (mmHg)	14 [3–28]	16 [6–26]	13 [6–26]	17 [8–26]*	13 [8–26]	16 [8–25]
CI (l min^-1^ m^-2^)	1.5 ± 0.4	1.4 ± 0.3	1.4 ± 0.3	1.5 ± 0.4	1.4 ± 0.3	1.4 ± 0.3
EF (%)	52 ± 12	50 ± 13	52 ± 12	46 ± 13*	51 ± 11	50 ± 13
∆ EF from before to during sternotomy (%)			1.4 ± 10	- 7.6 ± 16^†^		
SVRI	3176 ± 1251	3220 ± 873	3323 ± 1299	3868 ± 1062^§^	2918 ± 551	2992 ± 756
VO_2_ (ml min^-1^)	144 ± 25.4	155 ± 41.1	153 ± 34.2	186 ± 49.6*	156 ± 27	173 ± 41.1*
∆VO_2_ from before to during sternotomy (%)			- 0.4 ± 32	31 ± 46^†^		
S_v_O_2_	78.3 ± 6.6	78.3 ± 5.7	77.7 ± 5.9	78.6 ± 6.7	77.3 ± 9.2	79.0 ± 5.9

(mean ± standard deviation or median [range]).

*Abbreviations* and symbols: PAPsyst = systolic pulmonary artery pressure; PCWP = pulmonary capillary wedge pressure; CI = cardiac index; EF = left vetricular ejection fraction; SVRI = systemic vascular resistance index; VO_2_ = oxygen uptake; S_v_O_2_ = mixed venous oxygen saturation.

*p ≤ 0.05; ^†^p < 0.01; ^‡^p < 0.001 study group vs. control group; ^§^p ≤ 0.05 before vs. during sternotomy (paired *t*-test).

### Postoperative pain and opioid analgesic consumption

The median postoperative NRS scores on the day of surgery were 1.5 (range 0–5) in the study group and 2 (range 0–5) in the control group (p = 0.68). On the first day after surgery they were 2 (range 0–6) in the study group and 2.5 (range 0–8) in the control group (p = 0.1).

The median postoperative oxycodone consumption was 3 mg (range 0–26.5 mg) in the study group and 3 mg (range 0 – 19 mg) in the control group on the day of surgery (p = 0.64). The corresponding doses on the first day after surgery were 3 mg (range 0 – 23 mg) and 1.5 mg (range 0 – 15 mg) in the study and control groups, respectively, (p = 0.4).

## Discussion

The results of this study indicate that high-dose remifentanil given in addition to balanced anesthesia with high-dose sufentanil and sevoflurane can attenuate the increase in blood pressure and sympathetic nervous system activity induced by sternotomy and sternal spread.

Systolic and mean arterial blood pressures increased significantly in the control group but not in the study group. Six patients in the control group (20%) but only one in the remifentanil study group (3%) had a greater than 15% increase in systolic blood pressure.

The hemodynamic response rate of 20% in our control group is similar to that reported by other authors [5, 8, 23–25]. Philbin et al. observed hemodynamic responses rates up to 50% with sufentanil doses between 10 and 40 μg kg^-1^ body weight [8]. Thompson et al. using targeted sufentanil plasma concentrations between 3.0 ± 0.7 ng ml^-1^ and 7.1 ± 1.3 ng ml^-1^ found a greater than 20% increase in mean arterial pressure in 33% to 50% of their patients [24]. These corresponded to total sufentanil doses of 6.8 ± 1.1 μg kg^-1^ and 20.9 ± 2.0 μg kg^-1^, respectively. Ahonen et al. [23] observed a 20% hemodynamic response rate to sternotomy in patients with a sufentanil plasma concentration of 0.7 ng ml^-1^. No dose of sufentanil in any of these studies reduced the response rate to below 20%.

The total sufentanil dose was the same in both groups and was greater than that necessary to obtain full μ-receptor occupation and maximum ceiling effect. In our study, the total sufentanil dose administered during the study period was approximately 4.3 ± 0.5 μg kg^-1^, which corresponds to a plasma concentration in the range of 2 ng ml^-1^. This is lower than the dose employed in some studies of the effects of high-dose sufentanil on the response to sternotomy [8, 24], and one might argue that one would have obtained the observed effect of remifentanil by simply increasing the sufentanil dose. However, the results of dose-effect studies and the lack of increased effect of high-dose sufentanil contradict this assumption. The dose–response curve of sufentanil follows the typical sigmoid curve with a maximum ceiling effect between 1.25 and 1.4 ng ml^-1^ (see [8, 9]). The hemodynamic response rate to sternotomy in studies was between 20% and 60% in studies using sufentanil plasma concentrations greater than this concentration. This ceiling effect concentration is approximately double the *in vitro* binding affinity of sufentanil to the μ-opioid receptor [26] and corresponds to a total dose of approximately 2 μg kg^-1^ in patients.

Systemic vascular resistance, a parameter also linked to SNS activity, increased significantly in the control group during sternotomy and sternal spread. The observed simultaneous decrease in ejection fraction is possibly a result of the increased afterload. Stephan et al. described a similar response to sternotomy with increased SVR and reduced cardiac index in patients undergoing myocardial revascularization [27]. This response was not observed in our study group with remifentanil suggesting that remifentanil attenuates or suppresses SNS activation.

The electrodermal response (EDR) is the most direct non-invasive clinical monitor of sympathetic nervous system (SNS) activity. Increased activity stimulates secretion of eccrine sweat glands with a subsequent change in skin conductance [22, 28, 29]. The stimulus of intubation, which was performed before starting the remifentanil infusion, induced an EDR in an identical number of patients (responders) in the two groups. Remifentanil significantly reduced the number of patients with an electrodermal response to sternotomy and chest spreading by 50% indicating that it suppressed the typical activation of the SNS.

Increased oxygen uptake is evidence of sympathetic nervous system activation [30] and our data suggest that remifentanil suppresses the sympathetic response.

Plasma catecholamine concentrations are often used to quantify sympathetic nervous system activity but these would have given meaningless results in our study since many of the patients were receiving catecholamine infusions for circulatory support during the study period. These infusions were not responsible for the observed increase in oxygen consumption, since they were neither started nor the infusion rate altered in the period before or during sternotomy.

The inability of sufentanil to reduce the incidence of hemodynamic responders at doses that would assure a saturation of μ-opioid receptors suggests that the observed effect of remifentanil is not mediated by μ receptors. Remifentanil has a lower affinity for kappa and delta opioid receptors, but a considerable degree of receptor occupation would still have been attained at the plasma concentrations achieved with the high doses applied in this study. The binding affinity of remifentanil is reported to be 2.6 nM for the μ-opioid receptor and 66 nM for the delta receptor [13]. He and Lee showed that the delta opioid agonist DPDPE acts in a synergistic fashion to reduce the analgesic ED_50_ of the μ-receptor agonist DAMGO even though binding affinity of DPDPE to delta receptors is at least two orders of magnitude lower than that of DAMGO to μ receptors [31]. An early study using specific antagonists of delta opioids receptors (naltrindole and naltriben) suggests that sufentanil also has some affinity to delta receptors to produce respiratory depression [32]. Preconditioning mouse hearts with a remifentanil concentration of 10 nM (=3.8 ng ml^-1^) was sufficient to confer cardioprotection and reduce infarct size. This effect was blocked by naltrindol, a delta-opioid receptor antagonist [13]. But while a direct effect on the myocardium might explain the attenuated blood pressure increase, it would not explain the other observed effects on sympathetic nervous system activity. Whether the cardioprotective effects of remifentanil that were demonstrated in mice are relevant for cardiac surgery remains to be determined by further studies.

The depressant effects of remifentanil on hemodynamics have been shown to be reversed by atropine and are considered a stimulatory effect of remifentanil on the parasympathetic nervous system [33, 34]. These would counteract sympathetic stimulation, and the hemodynamic effects of remifentanil observed in the present study could be attributed to its vagomimetic actions. This hypothesis is supported by the course of the Surgical Pleth Index (SPI), which was used to guide the administration of remifentanil. The SPI is considered to be a measure of the balance between sympathetic and parasympathetic nervous system (PNS) activity. High intraoperative values correlate with stress [35], while lower values indicate a preponderance of PNS over SNS activity. The remifentanil dose in the study group was increased until the SPI was in an extremely low range corresponding to increased PNS activity.

Some clinicians avoid remifentanil for major surgery because of their concerns about opioid-induced hyperalgesia [14, 15]. This phenomenon is thought to result from remifentanil acting at delta-opioid receptors to enhance spinal N-methyl-D-aspartate receptor function [36]. In our study, the reported intensity of postoperative pain and the amounts of administered analgesics were the same in the groups with and without remifentanil. Lahtinen et al. [37] also did not find any evidence of opioid-induced hyperalgesia following cardiac surgery when remifentanil was combined with sufentanil.

These findings suggest that remifentanil does not induce clinically relevant hyperalgesia when given for a short period in combination with sufentanil.

## Conclusions

High-dose remifentanil added to balanced high-dose sufentanil-sevoflurane anesthesia can suppress the adrenergic response to sternotomy and sternal spread and attenuate increases in blood pressure, systemic vascular resistance and oxygen uptake. In this setting, remifentanil does not induce postoperative hyperalgesia.

## Electronic supplementary material

Additional file 1: CONSORT 2010 checklist of information to include when reporting a randomised trial*. (DOC 219 KB)
